# A 55-year-old man with mild shortness of breath

**DOI:** 10.1007/s12471-019-01312-0

**Published:** 2019-07-23

**Authors:** A. D. Egorova, J. M. Smit, P. Kiès

**Affiliations:** Department of Cardiology, Heart Lung Centre, Leiden University Medical Centre, Leiden, The Netherlands

A 55-year-old male was referred by his general practitioner due to mild complaints of shortness of breath (no angina) upon exertion. He used to work as a gym teacher at a secondary school and currently exercises intensively for up to 10 h a week. His father died suddenly at the age of 52 years while cross-country skiing. No autopsy was performed. The patient is being treated with perindopril 2 mg once a day due to hypertension and has a blood pressure of 140/90 mm Hg and a resting peripheral O_2_ saturation of 97% when breathing room air. Electrocardiography showed sinus rhythm with normal de- and repolarisation and a physical examination did not reveal any abnormalities. Transthoracic echocardiography showed normal biventricular and valvular function. Chest radiography revealed an abnormality and prompted further investigation by computed tomography (Fig. 1a–c).

**Fig. 1:**
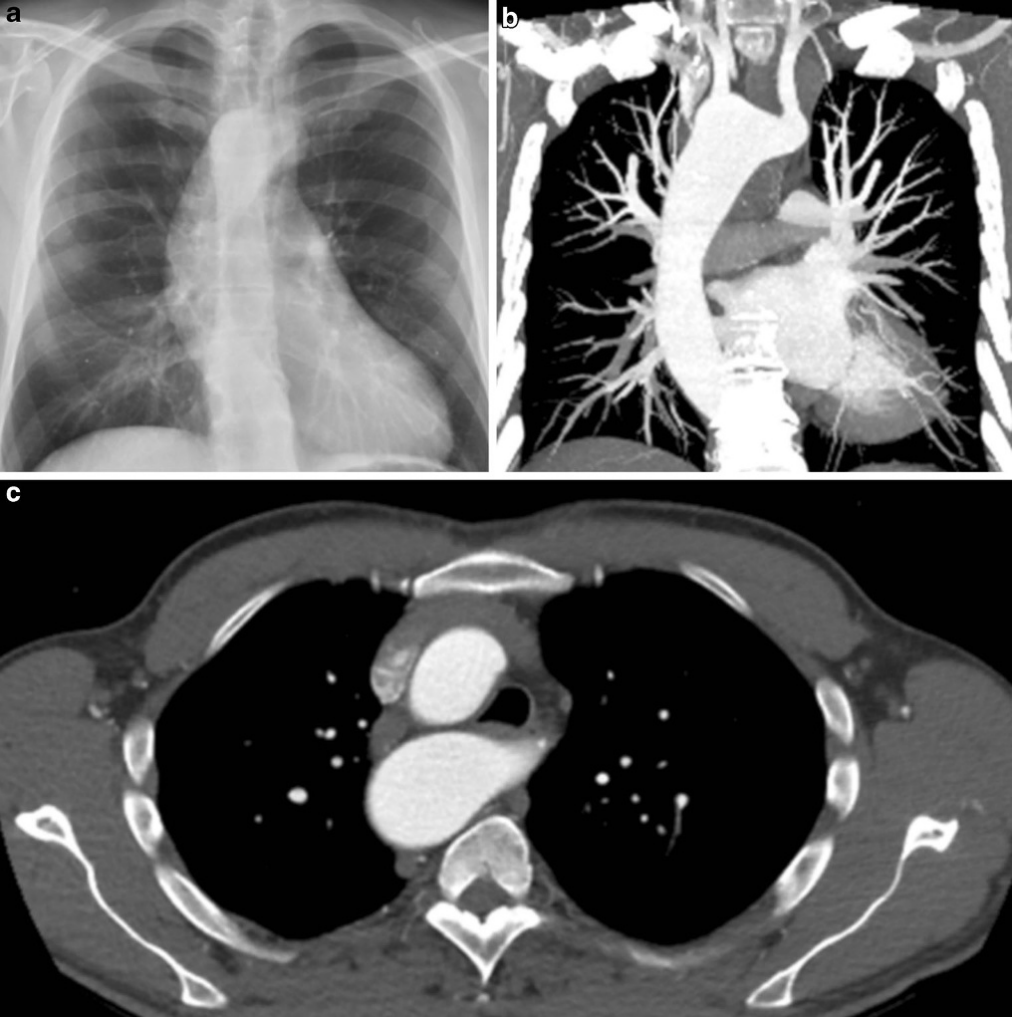
An anteroposterior chest radiograph (**a**) and the corresponding frontal plane (**b**) and transverse plane (**c**) CT images of the patient described

What is the most likely diagnosis?

A. Acute aortic syndrome
B. Persistent left superior vena cava
C. Right-sided aortic arch with an aberrant left subclavian artery
D. Double aortic arch

## Answer

You will find the answer elsewhere in this issue.

